# The Assessment of Knowledge, Attitude, and Practice of Paracetamol and Ibuprofen Administration Among Saudi Parents in the Makkah Region

**DOI:** 10.7759/cureus.67123

**Published:** 2024-08-18

**Authors:** Duaa N Alwashali, Refal T Abumansour, Aesha H Alansari, Turki A Alotaibi, Anwar A Zaki, Zayna A Fatani, Naif Al-Meqaty, Mohammed Ageel

**Affiliations:** 1 Medicine, Umm Al-Qura University, Makkah, SAU; 2 Pediatrics, Umm Al-Qura University, Makkah, SAU; 3 Pediatric Surgery, Umm Al-Qura University, Makkah, SAU

**Keywords:** childhood, practice, parents, attitude, knowledge, saudi arabia, ibuprofen, paracetamol, ibuprofen and paracetamol combination therapy

## Abstract

Introduction

Paracetamol and ibuprofen, widely used for pediatric fever and pain, are safe when administered correctly. However, the caregiver's lack of understanding poses risks such as overdose. Addressing knowledge gaps is crucial due to reported variations in over-the-counter medication practices. "Fever phobia" underscores parental anxiety, stressing the ongoing need for research in this healthcare domain.

Methodology

This is a descriptive cross-sectional design targeting Saudi parents and caregivers from the Makkah region who have children aged 0-10 years. Data was collected via a self-administered validated online questionnaire in the Arabic language using a convenient sampling technique. The data was cleaned in Excel and analyzed using SPSS version 29 (IBM Inc., Armonk, New York).

Results

Our study included 449 parents and caregivers in the Makkah Region, of whom 337 (75.1%) were female, 179 (39.9%) were aged 18-29, and 425 (94.7%) were Saudi nationals. Knowledge assessment revealed gaps; e.g., only 86 (26.6%) identified baby weight as a dosage factor. Attitudes varied, with 152 (47.1%) associating paracetamol/ibuprofen with liver harm. Logistic regression showed no significant predictors for high-level knowledge, positive attitudes, or good practices, except for gender-influencing good practices (p=0.035, aOR=1.839). Significantly, males exhibited better practices regarding using of paracetamol.

Conclusion

Our study highlights knowledge gaps among parents and caregivers in the Makkah Region regarding pediatric fever management with paracetamol and ibuprofen. Attitudes varied, and gender significantly influenced good practices, with males demonstrating better adherence to the proper practice of managing children using paracetamol and ibuprofen.

## Introduction

Paracetamol and ibuprofen are commonly used over-the-counter (OTC) medications for managing fever and pain in children. They are considered safe and effective when used appropriately. However, inappropriate administration, such as incorrect dosing or frequency, may lead to adverse effects, including drug toxicity or inadequate symptom control. Due to the widespread use of these medications, it is essential to assess the knowledge, attitude, and practice of paracetamol and ibuprofen administration among caregivers.

This research aims to evaluate parents' and caregivers' understanding of the appropriate use of these medications for their children and identify potential gaps in knowledge and misconceptions that may contribute to inappropriate use [1]. Parents play a critical role in managing their children's health and well-being, often making decisions about medication administration without consulting healthcare professionals. A lack of understanding about the proper use of paracetamol and ibuprofen may lead to unnecessary risks, including overdose, underdose, or adverse drug interactions. Previous studies have reported varying levels of knowledge and practice among parents regarding the administration of OTC medications, highlighting the need for ongoing research in this area [2].

Even though it is considered a natural defense mechanism, fever was the cause of 15-25% of pediatric emergency room and primary care visits [3]. The term "fever phobia" frequently describes worried parents with irrational anxiety about fever [4]. Therefore, many parents inappropriately self-administer anti-pyritic drugs, especially paracetamol [5]. A recent 2021 study was conducted in the city of Al-Riyadh, with a total of 571 participants. The study aimed to evaluate the attitudes and practices of Saudi parents towards self-medication using OTC medicines to treat their children. The results of the online questionnaire in this study showed that paracetamol was the most common OTC medication, with fever as the leading cause [6].

To the best of our knowledge, this study is the first to evaluate parents' and caregivers' knowledge, attitudes, and practices regarding the administration of paracetamol and ibuprofen in the Makkah Region. We aim to provide information on the level of knowledge and highlight the knowledge gap with the hope of lowering morbidity and mortality through enhancing caregivers' education and pediatric healthcare. Although several prior studies have revealed that parents often and routinely give their kids ibuprofen and paracetamol, caregivers frequently administer the wrong doses. In this study, we intend to evaluate parents' and caregivers' knowledge, attitude, and practice of paracetamol and ibuprofen administration among Saudi parents and caregivers in the Makkah Region due to the lack of data regarding caregivers management understanding and practice.

## Materials and methods

Study design and participants

This cross-sectional study used a self-administered validated online questionnaire on the general population in Makkah Region. Participants were recruited by submitting the survey and sending it through social media using the convenient sampling technique.

Participants included parents and caregivers who have children aged 0-10 years. The questionnaire was in Arabic. Informed consent was obtained electronically prior to the availability of the survey. Participants who refused to participate in the study or did not meet the eligibility requirements were excluded.

Ethical considerations and sample size

The study was conducted between August 2023 and January 2024. After obtaining IRB approval from Umm Al-Qura University's Biomedical Ethics Committee (Approval No.HAPO-02-K-012-2023-10-1775), the data were obtained via an online self-administered questionnaire in the Arabic language designed by Google Forms (Google, Mountain View, California) and distributed among the general population.

The target sample size was identified by OpenEpi version 3, and 384 participants were considered the appropriate sample size [7]. The overall sample size was increased to a maximum of 500 participants in case of potential data loss and to generalize the study results more efficiently.

Study tool and scoring

We used a validated self-administered questionnaire, which was obtained from a previously published study [8]. The questionnaire was generated in the Arabic language, and it is included in the Appendix. It comprised 27 questions divided into four sections. Written informed consent was required before answering the questions. The caregiver's demographic information was requested in the first section. The second section evaluated the parents' knowledge of using paracetamol and ibuprofen. The third section investigated the caregiver's perspective on using paracetamol and ibuprofen. Finally, the fourth section explored how the caregivers use paracetamol and ibuprofen. Several measures were taken to ensure the confidentiality of participants' information in this study.

Statistical analysis

The data were collected using an Excel sheet (Microsoft, Redmond, Washington). Data analysis was performed using Statistical Package for Social Sciences (SPSS) version 29 (IBM Corp., Armonk, NY, USA). Frequency and percentages were used for the descriptive analysis. Participants with scores below the median were considered to have poor knowledge, whereas those with scores equal to or above the median were considered to have good knowledge of the topic. We used a logistic regression model to analyze the sociodemographic predictors of caregivers' high knowledge levels. The significance level was 0.05. All data were anonymized.

## Results

Our study included 449 parents and caregivers (Table 1). Remarkably, 337 (75.1%) respondents were female, while 112 (24.9%) were male. Age distribution shows that the majority fell within the 18-29 years category 179 (39.9%), with considerable proportions in other age groups. Saudi nationals constituted 425 (94.7%) of the sample. Regarding education, 346 (77.1%) have a university degree, and 208 (46.3%) are employed full-time. Family size varied, with 133 (29.6%) having more than four children. Notably, 323 (71.9%) of caregivers have treated their child's high temperature with paracetamol or ibuprofen.

**Table 1:** Sociodemographic and other features of the caregivers

Variables		N(%) (n=449)
Gender	Female	337 (75.1%)
	Male	112 (24.9%)
Age	<18 years	12 (2.7%)
	18-29 years	179 (39.9%)
	30-39 years	83 (18.5%)
	40-59 years	156 (34.7%)
	60-65 years	14 (3.1%)
	>65 years	5 (1.1%)
Nationality	Non-Saudi	24 (5.3%)
	Saudi	425 (94.7%)
Education status	Primary/middle	19 (4.2%)
	Secondary	55 (12.2%)
	University	346 (77.1%)
	Post-graduate	29 (6.5%)
Employment status	Unemployed	180 (40.1%)
	Part-time	61 (13.6%)
	Full Time	208 (46.3%)
Number of children	No children	150 (33.4%)
	1 child	68 (15.1%)
	2 child	51 (11.4%)
	3 child	47 (10.5%)
	>4 child	133 (29.6%)
Ever treated a child's high temperature with paracetamol or ibuprofen?	No	126 (28.1%)
	Yes	323 (71.9%)

**Figure 1:**
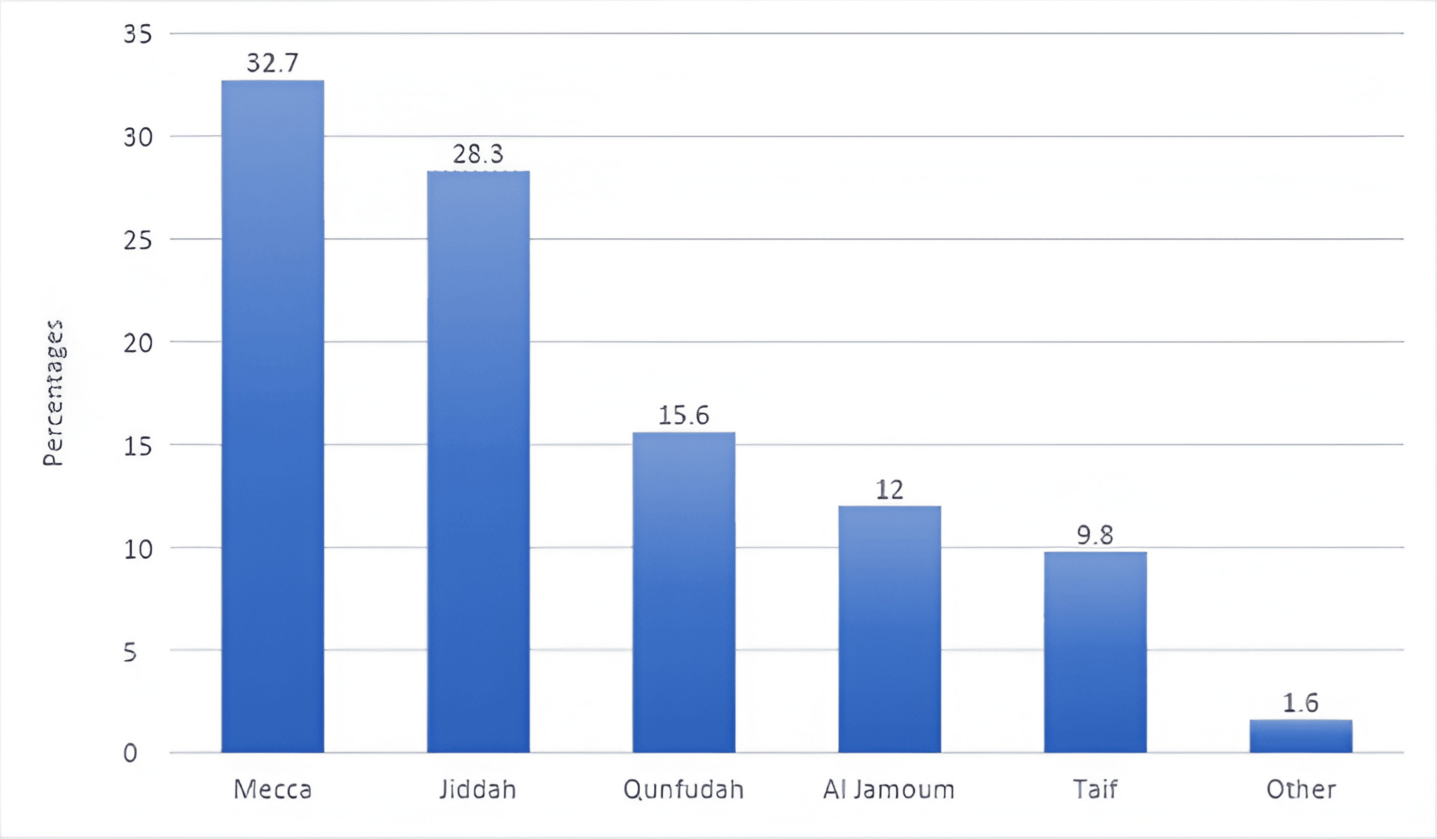
City-wise distribution of caregivers

Figure 1 shows the city-wise distribution of caregivers with Mecca being the most common city followed by Jeddah.

Table 2 shows the assessment of knowledge on paracetamol and ibuprofen use. Regarding factors influencing dosage selection, 86 (26.6%) correctly identified baby weight. For paracetamol, 114 (35.3%) were aware of the recommended dose (10-15 mg), and 138 (42.7%) knew the frequency of administration (every four or six hours). Only three (0.9%) correctly stated the maximum daily frequency for paracetamol (five times). Regarding ibuprofen, 142 (44.0%) knew the recommended dose (4-10 mg), and 127 (39.3%) correctly identified its frequency (every eight hours). Similarly, 129 (39.9%) were knowledgeable about the maximum daily frequency for ibuprofen (three times). Based on these questions, 133 (41.2%) of the users are above the median knowledge score, while 190 (58.8%) are below. Therefore, most had inadequate knowledge.

**Table 2:** Questions assessing the knowledge of caregivers regarding paracetamol/ibuprofen Use

Questions	Correct answers	N (%) (n=323)
Are you aware of the factors affecting the selection of doses (paracetamol and ibuprofen)	Baby weight	86 (26.6%)
Recommended dose of paracetamol	10 - 15 mg	114 (35.3%)
How often is paracetamol given	Every 4 or 6 hours	138 (42.7%)
Maximum daily frequency of administration of paracetamol	5 times	3 (0.9%)
Recommended dose of ibuprofen	4-10 mg	142 (44%)
How often is ibuprofen given	Every 8 hours	127 (39.3%)
Maximum daily frequency of administration of ibuprofen	3 times	129 (39.9%)
Knowledge level		
Knowledge level based on these questions	Low-level knowledge (below median score)	190 (58.8%)
	High-level knowledge (above median score)	133 (41.2%)

Table 3 shows a comprehensive assessment of the attitude and practice of caregivers regarding the use of paracetamol and ibuprofen. In terms of caregivers' attitudes, a significant portion is aware of the side effects, with 152 (47.1%) associating paracetamol and ibuprofen with liver harm and 148 (45.8%) with kidney harm. The majority seek dosage information from doctors 244 (75.5%) and pharmacists 176 (54.4%). When it comes to the form of administration, 245 (75.9%) prefer the liquid form. Notably, 264 (81.7%) believe it is incorrect to administer both medications simultaneously without consulting a doctor. In terms of caregiver practice, 236 (73.1%) measure a child's temperature using a thermometer, and 212 (65.6%) commonly use paracetamol as an antipyretic. Additionally, 297 (92.0%) measure a child's temperature before administering a fever reducer, while 252 (78.0%) make a point to read the attached leaflet.

**Table 3:** Assessment of attitude and practice of caregivers regarding paracetamol/ibuprofen use

Questions		N (%) (n=323)
Caregivers attitude		
Are you aware of the side effects of paracetamol and ibuprofen?	Harmful to the liver	152 (47.1%)
	Harmful to the kidneys	148 (45.8%)
	Allergic reactions	98 (30.3%)
	Harmful to the stomach	89 (27.5%)
	Don't know	14 (4.3%)
Where do you get information regarding the dosage of paracetamol and ibuprofen?	Doctor	244 (75.5%)
	Pharmacist	176 (54.4%)
	Social media/media	57 (17.6%)
	Family members	43 (13.3%)
What form of paracetamol or ibuprofen do you use?	Drink (paracetamol/ ibuprofen)	245 (75.9%)
	Syrup (paracetamol/ ibuprofen) & suppositories (paracetamol)	53 (16.4%)
	Suppositories (paracetamol)	22 (6.8%)
	By injection (paracetamol/ ibuprofen)	3 (0.9%)
Do you think it is correct to give paracetamol and ibuprofen without consulting a doctor?	No	190 (58.8%)
	Yes	133 (41.2%)
Do you think it is correct to give paracetamol and ibuprofen together?	No	264 (81.7%)
	Yes	59 (18.3%)
Caregivers practice		
At what temperature is an antipyretic given?	38 - 38.5 degrees	149 (46.1%)
	More than 38 degrees	127 (39.3%)
	More than 39 degrees	43 (13.3%)
	More than 40 degrees	4 (1.2%)
How do you measure a child's temperature?	Using a thermometer	236 (73.1%)
	By touching forehead	78 (24.1%)
	By touching lips	6 (1.9%)
	Other	3 (0.9%)
What is the most common antipyretic you use for your child?	Paracetamol	212 (65.6%)
	Ibuprofen	45 (13.9%)
	Both of them	61 (18.9%)
	Other	5 (1.5%)
What tools do you use to measure the dose of paracetamol and ibuprofen when giving them to a child?	Standard needle	107 (33.1%)
	Standard spoon	191 (59.1%)
	Teaspoon	23 (7.1%)
	Other	2 (0.6%)
Do you measure the child's temperature before giving him an antipyretic?	No	26 (8.0%)
	Yes	297 (92.0%)
Do you usually make sure to read the leaflet attached to the medicine?	No	71 (22.0%)
	Yes	252 (78.0%)
Do you give paracetamol and ibuprofen for reasons other than fever?	No	186 (57.6%)
	Yes	137 (42.4%)

Table 4 shows the results of a logistic regression model assessing predictors of high-level knowledge among caregivers regarding the use of paracetamol and ibuprofen. Age (p=0.924, aOR=1.010, 95% CI: 0.820-1.245), gender (p=0.517, aOR=1.195, 95% CI: 0.697-2.047), nationality (p=0.650, aOR=0.797, 95% CI: 0.298-2.128), and higher education (p=0.912, aOR=0.977, 95% CI: 0.642-1.485) did not significantly predict high-level knowledge. Employment status, with unemployed as the reference, showed no significant differences for part-time (p=0.540, aOR=1.262, 95% CI: 0.600-2.656) or full-time jobs (p=0.283, aOR=1.368, 95% CI: 0.772-2.422). The number of children (p=0.460, aOR=1.075, 95% CI: 0.887-1.302) also did not emerge as a significant predictor.

**Table 4:** Predictors of high-level knowledge among caregivers (logistic regression model) B - The regression slope, or unstandardized coefficient; Sig. - p-value (statistically significant p-value is less than 0.05); ExpB - odds ratio; CI - confidence interval

Variables	B	Sig.	Exp(B)	95% CI	
				Lower	Upper
Age	.010	.924	1.010	.820	1.245
Gender (male)	.178	.517	1.195	.697	2.047
Nationality (Saudi)	-.227	.650	.797	.298	2.128
Higher education	-.024	.912	.977	.642	1.485
Employment (unemployed)	Ref	.552	Ref	Ref	Ref
Employment (part-time job)	.233	.540	1.262	.600	2.656
Employment (full-time job)	.313	.283	1.368	.772	2.422
Number of children	.072	.460	1.075	.887	1.302
Constant	-.533	.408	.587		

Table 5 shows that age (p=0.313, aOR=0.898, 95% CI: 0.729-1.107), gender (p=0.842, aOR=0.946, 95% CI: 0.546-1.639), and higher education (p=0.379, aOR=1.207, 95% CI: 0.794-1.834) did not significantly predict positive attitudes. However, Saudi nationality showed a trend (p=0.174), with an odds ratio of 2.113 (95% CI: 0.718-6.219). Employment status, whether unemployed (p=0.546), part-time (p=0.748), or full-time (p=0.275), did not emerge as significant predictors. The number of children also did not significantly predict positive attitudes (p=0.352).

**Table 5:** Predictors of positive attitude among caregivers (logistic regression model) B - The regression slope, or unstandardized coefficient; Sig. - p-value (statistically significant p-value is less than 0.05); ExpB - odds ratio; CI - confidence interval

Variables	B	Sig.	Exp(B)	95% CI	
				Lower	Upper
Age	-.108	.313	.898	.729	1.107
Gender (male)	-.056	.842	.946	.546	1.639
Nationality (Saudi)	.748	.174	2.113	.718	6.219
Higher education	.188	.379	1.207	.794	1.834
Employment (unemployed)	Ref	.546	Ref	Ref	Ref
Employment (part-time job)	-.121	.748	.886	.425	1.850
Employment (full-time job)	-.314	.275	.730	.416	1.283
Number of children	-.091	.352	.913	.755	1.105
Constant	-.649	.338	.523		

Table 6 shows the results of a logistic regression model examining predictors of good practices among caregivers concerning the use of paracetamol and ibuprofen. Age (p=0.986, aOR=1.002, 95% CI: 0.801-1.254) and higher education (p=0.341, aOR=0.809, 95% CI: 0.523-1.252) did not significantly predict good practices. However, gender (p=0.035, aOR=1.839, 95% CI: 1.045-3.235) showed significance, indicating that males were more likely to exhibit good practices. Nationality (p=0.198) displayed a trend towards significance, with an odds ratio of 2.328 (95% CI: 0.644-8.419). Employment status, whether unemployed (p=0.484), part-time (p=0.230), or full-time (p=0.730), did not emerge as significant predictors. The number of children also did not significantly predict good practices (p=0.827).

**Table 6:** Predictors of good practice among caregivers (logistic regression model) * - statistically significant p-value (less than 0.05); B - The regression slope, or unstandardized coefficient; Sig. - p-value (statistically significant p-value is less than 0.05); ExpB - odds ratio; CI - confidence interval

Variables	B	Sig.	Exp(B)	95% CI	
				Lower	Upper
Age	0.002	0.986	1.002	0.801	1.254
Gender (male)	0.609	.035*	1.839	1.045	3.235
Nationality (Saudi)	0.845	0.198	2.328	0.644	8.419
Higher education	-.212	0.341	0.809	0.523	1.252
Employment (unemployed)	Ref	.484	Ref	Ref	Ref
Employment (part-time job)	-.525	0.230	0.592	0.251	1.394
Employment (full-time job)	-.106	0.730	0.900	0.493	1.641
Number of children	0.023	0.827	1.023	0.833	1.257
Constant	-1.301	0.092	0.272		

## Discussion

Paracetamol and ibuprofen, commonly used for pediatric fever and pain, are safe when appropriately administered. Furthermore, a study was done in 2021 in Saudi Arabia on a total of 656 parents, aimed to assess their knowledge, beliefs, and behavior toward fever and its management. The study used an online questionnaire, which revealed that the most common antipyretic drugs used among parents were paracetamol, followed by antibiotics and ibuprofen [9]. Similarly, Chiappini et al. (2023) show a significant reduction in body temperature one hour after administration of paracetamol and the absence of fever in almost all the children included in the study [10]. Moreover, Hay et al. (2008) concluded that for unwell children with a fever, start with ibuprofen; weigh the risks of exceeding paracetamol and ibuprofen doses against the benefits (2.5 hours fever reduction) [11]. In 2021, another study was done in Jeddah, assessing the knowledge, attitude, and practice of paracetamol and ibuprofen administration among 493 caregivers. The results indicated that most caregivers had inadequate knowledge regarding antipyretic administration, and half of the participants were unsure about the correct dosage [8]. Moreover, a study in 2015 on 1272 pediatric patients in Al-Dammam Maternal and Child Hospital shed light on the problem of accidental poisoning among children, its factors, and epidemiology. Unfortunately, the results showed that the most common presentation was medication toxicity, and paracetamol was at the top of the list of medications [12]. The caregiver's lack of understanding in this literature was demonstrated in the administration method and even in attaining the degree of toxicity [8,12].

Parents, often decision-makers in their children's health, may lack understanding, leading to risks such as overdose or inadequate symptom control. Addressing gaps in caregivers' knowledge is essential, given the reported variability in OTC medication administration practices. The term "fever phobia" highlights caregivers' anxiety, emphasizing the need for ongoing research in this critical healthcare area [13]. Thus, the findings from our study provide valuable insights into the behaviors and perceptions of caregivers regarding the use of these commonly administered medications.

Our study's demographic profile aligns with existing literature to a considerable extent. The predominance of females among the surveyed caregivers (75.1%) is consistent with studies highlighting the central role of mothers in childcare decisions. The prevalence of mothers seeking healthcare information is well-documented, underscoring the need for targeted educational interventions aimed at enhancing their knowledge and practices concerning medications for children. However, a previous study by Keizer et al. (2020) showed that both fathers and mothers equally share childcare responsibilities for children's cognitive and body development [14]. Moreover, Roeters et al. (2016) showed that mothers find caring for a minor child to be meaningful while caring for an adolescent to be stressful. Fathers stress over infant care but find middle childhood care meaningful [15]. Notably, the distribution of age groups in our study echoes the trend observed in similar research, emphasizing the relevance of assessing the younger age cohort (18-29 years) due to their prominent representation (39.9%). This group may have unique needs and preferences that should be considered when designing educational materials and interventions.

Significantly, our study's knowledge assessment revealed gaps in caregivers' understanding, consistent with prior research, which shows that a low level of knowledge increases the risk of improper drug intake [8]. The identification of baby weight as a factor influencing dosage selection by only 26.6% of caregivers is a concerning finding. Similarly, Pan et al. (2016) show that the weight effect may be minimal, or the proper dosage can only be determined when weight is combined with other factors [16]. This aligns with studies emphasizing the importance of tailoring educational efforts to address specific knowledge deficits, such as accurate dosage calculations based on weight.

The low awareness (0.9%) of the maximum daily frequency of paracetamol administration (five times) is a critical point, highlighting the need for clearer communication on dosage limits. A previous study by Ramanayake et al. (2012) shows that eighty percent followed the correct (four times per day) dosing frequency [17]. Similarly, the knowledge gaps related to ibuprofen, including recommended dose and frequency, suggest a need for focused educational campaigns targeting these specific aspects.

Caregivers' attitudes and practices provide valuable insights into their decision-making processes. The high percentage (81.7%) opposing the simultaneous administration of paracetamol and ibuprofen without consulting a doctor reflects a positive aspect of caregivers' caution. Parri et al. (2023) show that the fixed-dose combination enhances efficacy and safety, ensuring better control of paracetamol and ibuprofen doses and minimizing incorrect dosage risks [18]. This aligns with existing recommendations and reflects the potential impact of health education campaigns emphasizing responsible medication use.

Remarkably, the predominant reliance on healthcare professionals, especially doctors, for dosage information (75.5%) underscores the pivotal role healthcare providers play in shaping caregivers' practices. Moreover, previous research shows that caregivers' main source of information on fever and paracetamol is their general practitioner [19]. However, the relatively lower percentage seeking information from pharmacists, social media, or family members suggests avenues for expanding educational outreach through multiple channels, which can increase caregivers' knowledge and lead to safer medication practices [20].

Interestingly, 92.0% of caregivers measure their child's temperature before administering a fever reducer which is reassuring and indicates a generally responsible approach. Similarly, AlAteeq et al. (2018) show that most of the caregivers (82%) touch their children to confirm fever before administration [21]. Nonetheless, the 8.0% who do not adhere to this practice highlight the importance of emphasizing temperature monitoring as a fundamental step in determining the need for antipyretic treatment.

Moreover, the logistic regression models explore potential predictors of high-level knowledge, positive attitudes, and good practices among caregivers. While demographic factors like age, gender, nationality, education, and employment did not consistently emerge as significant predictors, gender showed significance in predicting good practices. Males were more likely to exhibit good practices, indicating a gender-based variation that warrants further investigation. Similarly, in a previous study, 52.4% of fathers showed good practice as compared to 40.6% of mothers regarding fever management in children [22]. Moreover, cultural factors such as beliefs and traditions play a crucial role in healthcare decision-making, and understanding these dynamics can inform culturally sensitive health education initiatives [23].

Limitations

Limitations of our study include a potential response bias due to self-reporting, limited generalizability to diverse populations, and the reliance on cross-sectional data, which restricts the establishment of causal relationships. Moreover, our research was carried out in the Makkah region with a small sample size. So, we encourage more research among Saudi citizens with a larger sample size.

In addition, questions regarding socioeconomic status, caregivers' occupation, additional information about who administers the medication, and whether caregivers have experienced health education can reveal crucial data.

## Conclusions

Our study contributes valuable insights into the knowledge, attitude, and practices of Saudi parents and caregivers regarding paracetamol and ibuprofen administration. While aligning with existing literature, our findings emphasize the need for targeted interventions addressing specific knowledge gaps and cultural considerations. Tailoring educational initiatives that provide clear and concise information about the appropriate use of these medications, as well as guidelines for the safe administration and potential side effects, to the unique needs of caregivers, particularly those in the younger age group, can enhance the safe and effective use of these common medications for children.

## Appendices

Caregivers' demographic characteristics

Age of the caregiver:

·        <18 ·        18-29 ·        30-39 ·        40-59 ·        60-65 ·        >65

Sex

·        Male ·        Female

Nationality

·        Saudi ·        Non Saudi

Residence

·        Makkah ·        Other

Education level

·        Non-educated ·        Elementary ·        Intermediate ·        Secondary ·        University ·        Post graduated

Employment status

·        Not working ·        Part-time ·        Full time

Number of children

·        0 ·        1 ·        2 ·        3 ·        More than 3

Do you ever give your child paracetamol/ Ibuprofen when their temperature is high?

·        Yes ·        No

Caregivers' knowledge of antipyretic administration

Do you know the factors influencing the paracetamol/ ibuprofen dose?

·        Child's weight (correct answer) ·        Child's age ·        Illness severity (body temperature in °C)

What is the recommended dose for paracetamol?

·        Less than 10-15mg/kg/dose ·        10-15mg/kg/dose (correct answer) ·        More than 10-15mg/kg/dose

How often do you administer paracetamol?

·        Every 4 or 6 hours (correct answer) ·        Every 8 hours ·        Whenever the body temperature exceeds 37°C

What is the maximum daily frequency of paracetamol administration (times)?

·        2 ·        3 ·        4 ·        5 (correct answer) ·        6

What is the recommended dose for ibuprofen?

·        Less than 4-10mg/kg/dose ·        4-10mg/kg/dose (correct answer) ·        More than 4-10mg/kg/dose

How often do you administer ibuprofen?

·        Every 4 hours ·        Every 6 hours ·        Every 8 hours (correct answer) ·        Whenever the body temperature exceeds 37°C

What is the maximum daily frequency of ibuprofen administration (times)?

·        2 ·        3 (correct answer) ·        4 ·        5 ·        6

Caregivers' awareness of antipyretic administration

Are you aware of paracetamol's side effects?

·        Liver damage ·        Kidney damage ·        Allergy ·        Effect on stomach ·        Other

From where do you get information regarding paracetamol and ibuprofen?

·        Doctor ·        Pharmacist ·        Media ·        Family ·        Other

What is the form of paracetamol/ibuprofen that you use?

·        Syrups ·        Suppositories ·        Injection ·        Syrups and suppositories combined

Do you think it is fine to give paracetamol/ibuprofen to your child without a prescription?

·        Yes ·        No

Do you think it is fine to give paracetamol/ibuprofen together?

·        Yes ·        No

Caregiver practices in antipyretic administration

At which body temperature do you administer an antipyretic?

·        38-38.5 ·        More than 38.5 ·        More than 39 ·        More than 40

How do you measure the child’s body temperature?

·        Using a thermometer ·        By touching the forehead ·        By touching the lips ·        Other

The most common antipyretic that you administer to your child?

·        paracetamol ·        Ibuprofen ·        Both ·        Other

What do you use as a measuring device when you administer paracetamol/ibuprofen?

·        Teaspoon ·        Measuring spoon ·        Measuring syringe ·        Other

Do you measure the child's body temperature using a thermometer before administering antipyretics?

·        Yes ·        No

Do you read the medication leaflet?

·        Yes ·        No

Do you administer paracetamol/ibuprofen for reasons other than fever?

·        Yes .        No

القسم الأول : البيانات الديموغرافية المتعلقة بالوالدين

 س١ : العمر ؟

               ⁃             أقل من ١٨ سنة ⁃             من ١٨ إلى ٢٩ سنة ⁃             من ٣٠ إلى ٣٩ سنة ⁃             من ٤٠ إلى ٥٩ سنة ⁃             من ٦٠ إلى ٦٥ سنة ⁃             أكبر من ٦٥ سنة

س٢ : الجنس ؟

               ⁃             ذكر ⁃             أنثى

س٣: الجنسية ؟

               ⁃             سعودي/ة ⁃             غير سعودي/ة

س٤: السكن ؟

               ⁃             داخل مدن منطقة مكة ⁃             خارج مدن منطقة مكة

س٥: المستوى التعليمي ؟

               ⁃             غير متعلم ⁃             ابتدائي ⁃             متوسط ⁃             ثانوي ⁃             جامعي ⁃             دراسات عليا

س٦ : الحالة الوظيفية ؟

               ⁃             عاطل ⁃             دوام جزئي ⁃             دوام كلي

س٧: عدد الأطفال ؟

               ⁃             لايوجد ⁃             ١ طفل ⁃             طفلين ⁃             ٣ أطفال ⁃             أكثر من ٣ أطفال

س٨: هل سبق وتعاملت مع حالة ارتفاع درجة حرارة طفلك (الحمى) بإستخدام الباراسيتامول أو الأيبوبروفين ؟ً

               ⁃             نعم ⁃             لا

القسم الثاني : مدى معرفة الوالدين عن استخدام أدوية الباراسيتامول والأيبوبروفين

س١ : هل أنت مدرك/ة بالعوامل المؤثرة في اختيار جرعات ( الباراسيتامول والأيبوبروفين )

               ⁃             وزن الطفل (الاجابة الصحيحة) ⁃             عمر الطفل ⁃             شدة المرض ( بالإعتماد على درجة الحرارة )

س٢ : ماهي الجرعة الموصى بها للباراسيتامول ؟

               ⁃             أقل من ١٠-١٥ ميليجرام ⁃             ١٠-١٥ ميليجرام  (الاجابة الصحيحة) ⁃             أكثر من ١٠-١٥ ميليجرام

س٣ : كم عدد المرات التي يتم فيها إعطاء الباراسيتامول ؟

               ⁃             كل ٤ ساعات أو كل ٦ ساعات  (الاجابة الصحيحة) ⁃             كل ٨ ساعات ⁃             عندما تزيد درجة الحرارة عن ٣٧ درجة

س٤ : ماهو الحد الأقصى اليومي لتكرار إعطاء الباراسيتامول ؟

               ⁃             مرتين ⁃             ٣ مرات ⁃             ٤ مرات ⁃             ٥ مرات  (الاجابة الصحيحة) ⁃             ٦مرات

س٥ : ماهي الجرعة الموصى بها للأيبوبروفين ؟

               ⁃             أقل من ٤-١٠ ميليجرام ⁃             ٤-١٠ ميليجرام  (الاجابة الصحيحة) ⁃             أكثر من ٤-١٠ ميليجرام

س٦: كم عدد المرات التي يتم فيها إعطاء الأيبوبروفين ؟

               ⁃             كل ٤ ساعات ⁃             كل ٦ ساعات ⁃             كل ٨ ساعات  (الاجابة الصحيحة) ⁃             عندما تزيد درجة الحرارة عن ٣٧ درجة

س٧: ماهو الحد الأقصى اليومي لتكرار إعطاء الأيبوبروفين ؟

               ⁃             مرتين ⁃             ٣ مرات  (الاجابة الصحيحة) ⁃             ٤ مرات ⁃             ٥ مرات ⁃             ٦ مرات

القسم الثالث : توجه الوالدين فيما يتعلق بأدوية الباراسيتامول والأيبوبروفين

س١: هل أنت مدرك /ة بالأعراض الجانبية للباراسيتامول والأيبوبروفين ؟

               ⁃             مضر بالكبد ⁃             مصر بالمعدة ⁃             مضر بالكلية ⁃             الحساسية ⁃             غير ذلك

س٢: من أين تحصل /ين على المعلومات المتعلقة بجرعة ( الباراسيتامول والأيبوبروفين ) ؟

               ⁃             الطبيب ⁃             الصيدلي ⁃             وسائل التواصل الاجتماعي/ الإعلام ⁃             أفراد من العائلة ⁃             غير ذلك

س٣: ماهو الشكل الدوائي للباراسيتامول والأيبوبروفين التي تستخدمها ؟

               ⁃             شراب ⁃             تحاميل ⁃             عن طريق الحقن ⁃             شراب وتحاميل معاً

س٤ : هل تعتقد/ين أنه من الصحيح إعطاء ( الباراسيتامول والأيبوبروفين ) بدون إستشارة الطبيب ؟

               ⁃             نعم ⁃             لا

س٥: هل تعتقد/ين أنه من الصحيح إعطاء ( الباراسيتامول والأيبوبروفين ) معاً ؟

               ⁃             نعم ⁃             لا

القسم الرابع : ممارسات الوالدين المتعلقة بإعطاء أدوية الباراسيتامول والأيبوبروفين

س١ : عند أي درجة حرارة يتم إعطاء خافض الحرارة ؟

               ⁃             ٣٨-٣٨.٥ درجة ⁃             أكثر من ٣٨ درجة ⁃             أكثر من ٣٩ درجة ⁃             أكثر من ٤٠ درجة

س٢: كيف تقيس/ين درجة حرارة الطفل ؟

               ⁃             عن طريق الثيرمومتر ( جهاز لقياس الحرارة ) ⁃             عن طريق لمس الجبين ⁃             عن طريق لمس الشفاة ⁃             غير ذلك

س٣: ماهو أكثر خافض حرارة تستخدمه لطفلك ؟

               ⁃             باراسيتامول ⁃             أيبوبروفين ⁃             كلاهما ⁃             غير ذلك

س٤: ماهي الأدوات التي تستخدمها لقياس جرعة الباراسيتامول والأيبوبروفين عند إعطائها للطفل ؟

               ⁃             ملعقة قياسية ⁃             إبرة قياسية ⁃             ملعقة شاي ⁃             غير ذلك

س٥: هل تقوم بقياس درجة حرارة الطفل قبل إعطائه خافض الحرارة ؟

               ⁃             نعم ⁃             لا

س٦: هل تحرص/ين عادة بقراءة المنشور المرفق بالدواء ؟

               ⁃             نعم ⁃             لا

س٧: هل تقوم بإعطاء الباراسيتامول والأيبوبروفين لأسباب أخرى( لغير الحمى) ؟

               ⁃             نعم -             لا
